# Psychometric Properties of the Urdu Translation of Berg Balance Scale in People with Parkinson’s Disease

**DOI:** 10.3390/ijerph19042346

**Published:** 2022-02-18

**Authors:** Muhammad Kashif, Ashfaq Ahmad, Muhammad Ali Mohseni Bandpei, Syed Amir Gilani, Humaira Iram, Maryam Farooq

**Affiliations:** 1 University Institute of Physical Therapy, Faculty of Allied Health Sciences, University of Lahore, Lahore 42000, Pakistan; mohseni_bandpei@yahoo.com (M.A.M.B.); profgilani@gmail.com (S.A.G.); 2 Riphah College of Rehabilitation and Allied Health Sciences, Riphah International University, Faisalabad 38000, Pakistan; huumairaa15@gmail.com (H.I.); maryamfarooq50@gmail.com (M.F.)

**Keywords:** Berg Balance Scale, cross-cultural validation, outcome assessment, Parkinson’s disease, reliability

## Abstract

Background: The most common assessment tool used in clinical settings to detect changes in balance performance is the Berg Balance Scale (BBS). Thus, the purpose of this study was to translate the BBS into Urdu and investigate the psychometric properties (acceptability, internal consistency reliability, interrater reliability, construct validity) for individuals with Parkinson’s disease (PD). Methods: Eighty patients of either gender with idiopathic Parkinson’s disease, stages I–III on the modified Hoehn–Yahr (H&Y) scale, with intact cognition according to the Mini Mental Score Examination (MMSE) score (greater than or equal to 24) and independent of transfers, were included in this study. The BBS was translated according to international guidelines based on forward and backward translation processes. The test-retest reliability as well as intra- and inter-observer reliability was assessed by calculating the intra-class correlation coefficient (ICC). The internal consistency of the entire BBS score was assessed by calculating Cronbach’s α. The convergent validity was assessed by correlating the scale with the Unified Parkinson Disease Rating Scale (UPDRS) parts II and III and the Activity-specific Balance Confidence Scale (ABCS). The construct validity was assessed using a factor analysis. Results: The mean age of the subjects was 62.35 ± 5.74 in years (range: 60–87 years). The ICC for intra- and inter-observer reliability was 0.95 (*p* < 0.0001) and 0.99 (*p* < 0.001), respectively. Cronbach’s α was calculated as 0.81, which showed acceptable internal consistency of the Urdu version of the BBS. The test-retest reliability (ICC) of the Urdu version of the BBS was determined as 0.97 for the total score, and ranged from 0.66–0.95 for individual items. In terms of validity, the Urdu version of the BBS was correlated with the ABCS (in the positive direction) and UPDRS-II and III (in the negative direction) (r = 0.53, *p* < 0.001; r = −0.68, *p* < 0.001, r = −0.78, *p* < 0.0001), respectively. Conclusion: The Urdu version of the BBS is a reliable and valid scale to be used in balance assessment of population diagnosed with PD with excellent psychometric properties.

## 1. Introduction

Parkinson’s disease (PD) is a pathological condition characterised by a variety of motor as well as non-motor problems. Resting tremors, bradykinesia, rigidity, and postural instability are the main problems associated with motor symptomatology [1]. Its onset is more prevalent at 65 years of age or above and is a cause of emotional as well as financial stress on caregivers [2]. In the past 26 years, this problem has doubled in size worldwide, increasing from 2.5 million persons afflicted with the disease in 1990 to 6.1 million people reported to be afflicted with PD in 2016. This increase is likely related to longer life expectancies enabled by better health care outcomes, ultimately leading to an increase in the aging population [3].

Balance impairments are common among patients presenting with many of the neurological disorders, of which one is PD [4]. Once balance worsens in patients with PD, the healthcare professionals need a quantifiable tool to measure these changes and select a suitable treatment [5]. A systematic assessment of the balance impairments is also important in PD for the development of an efficient plan of care and evaluation of the efficacy of the rehabilitation protocol targeted at improving the balance as well as motor function over a specified period of time [6].

The Berg Balance Scale (BBS) was originally developed in 1989 by Katherine Berg to detect balance impairments in the elderly [7,8] but later on, the tool was standardised for PD as well as for stroke and similar neurological conditions [8,9]. The BBS is a short assessment tool that is often used to measure balance and mobility, as well as to find people who are at risk of falling [10,11,12]. The tool includes 14 different balance-related tasks, such as sitting, standing, and transferring. Each component is rated on a scale of 0 to 4, with 0 denoting severe impairment and 4 denoting the patient’s normal functioning balance system. The BBS components assess the patient’s ability to shift between different postures as well as their individual posture [8]. The total score ranges from 0 to 56 with a higher score indicating less balance impairment. A score less than 45 indicates the risk of falling, whereas a score of 56 shows good functional balance [1]. According to other criteria, patients with restricted mobility or who are wheel-chair bound, people who require assistance during the gait, and those independent in gait fall within the range of 0–20, 21–40, and 41–56 BBS score, respectively [7]. 

The BBS has been translated into many languages, including Persian, Turkish, and Russian, as well as Korean, Norwegian, and Japanese, due to its widespread use, and each version has proven to be reliable and valid [13,14,15,16,17,18]. The BBS is relatively safe and easy to use, and it has good inter- and intra-rater reliability in a wide range of patients, including those who have had brain injuries, strokes, or are old. Similarly, BBS has been shown to have high inter-rater and intra-rater reliability in PD patients [7,19,20,21,22]. The psychometric properties of BBS translation in different languages have been evaluated in people with Parkinson’s disease [23,24,25,26]. However, no study reported the psychometric properties of the Urdu translation for patients with PD. More than 65 million people worldwide speak Urdu, mostly from Pakistan and India. Moreover, Urdu is the national language of Pakistan [27,28]. In Pakistan, people do not read or understand English very well, so different questionnaires should be translated into Urdu. Therefore, the current study was aimed at determining the psychometric properties of the Urdu translation of BBS (BBS-U) in the context of its acceptability, internal consistency, reliability, interrater reliability, and construct validity among patients with PD.

## 2. Materials and Methods

This cross-sectional study was conducted from July to August 2020. Patients with a diagnosis of PD by the neurologist were recruited from the neurology and neurosurgical departments of tertiary hospitals in Faisalabad. The patients were then referred to the Department of Physical Therapy at Safi Hospital, where they were further evaluated for study eligibility by the Principal Investigator. Patients with PD who were being treated at the outpatient physical therapy department of Safi Hospital were also included in this study. PD patients with score ≥ 24 on Mini-Mental State Examination (MMSE) specifying intact cognition were included. MMSE is useful in detecting cognitive deterioration in patients with PD [29]. Patients with other neurological or orthopedic pathologies, visual and auditory impairments that can impair their balance ability, medications known to affect balance, severe pain (visual analogue scale > 75 mm) [30], amputations of the lower extremities, and unwillingness to participate in the study were excluded. The basic demographic information was recorded on the initial visit. Moreover, Parkinson’s specific tools including, modified (H&Y), UPDRS parts II and III, and ABCS were used and the findings were recorded. During this period, the patients were not given any type of treatment. To avoid any unexpected changes in motor symptoms, participants with PD were examined under the same conditions, i.e., during the “on” period [18]. 

### 2.1. Translation Procedure

**Figure 1:**
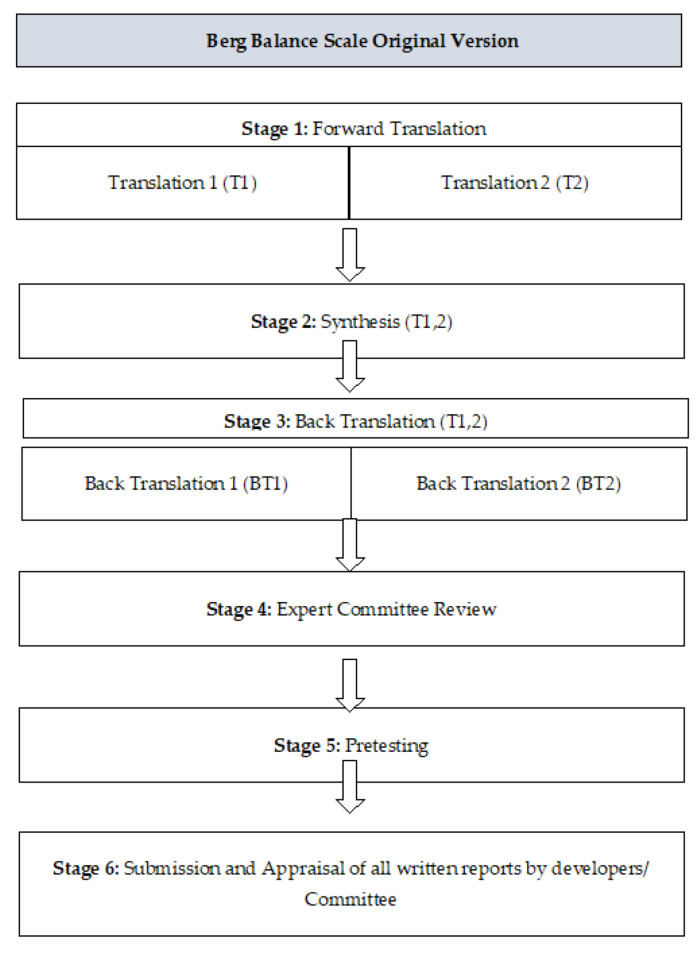
Stages of cross-cultural adaptation.

The approval was taken via email from Katherine Berg for the Urdu translation of BBS. The translation and the cross-cultural modification of BBS in the Urdu language were executed using six steps in compliance with previously published guidelines and in accordance with the criteria of consensus-based standards for the selection of health status measurement instrument (COSMIN) [31] (Figure 1).

#### 2.1.1. Stage 1: Translation

The translation of BBS was done by two native Urdu-speaking Pakistani translators. One of these bilingual and experienced translators was a physiotherapist who was aware of the concept of this study, and the other was a translation expert with a non-medical or clinical background. Both translators independently translated the BBS into the Urdu language and provided a written translated report of the scale based on the conceptual framework of the original scale rather than literal conversion. At the end of this step, two translated versions of the scale, translation 1 (T1) and translation 2 (T2), were obtained. 

#### 2.1.2. Stage 2: Synthesis

During this step, the original version of the scale along with translations 1 (T1) and 2 (T2) were synthesized to obtain a common translation version (T-12). The translation was then composed according to the English version of BBS. All the points were made easy to understand by using the comprehensible meanings of the words translated into Urdu. The full Urdu translation of BBS (T-12) was completed at this stage.

#### 2.1.3. Stage 3: Back Translation

At this stage, the back translation of the Urdu-translated version into the English language was managed. For this purpose, the help of two bilingual translators was taken. Both of these translators were native Pakistanis and had a great command of the Urdu and English languages, with English as their mother tongue. They were not familiar with the purpose of this study or the original English version of BBS. After the complete back translation, the translators provided the resultant copies entitled as “BT1” and “BT2”.

#### 2.1.4. Stage 4: Expert Committee Review

The committee comprised methodologists, health professionals, language professionals, and the translators (forward and back translators) involved in the process up to this point. The expert committee’s role was to consolidate all the versions of the questionnaire and develop what would be considered the pre-final version of the questionnaire for field testing. The material at the disposal of the committee included the original questionnaire, and each translation (T1, T2, T12, BT1, and BT2), together with corresponding written reports (which explained the rationale of each decision at earlier stages). As the committee was already aware of the purpose of the study, they compared both of the versions. After comparing the Urdu and English versions of the scale, it was further updated and edited by the members of the committee.

#### 2.1.5. Stage 5: Pretesting

The pre-final translated version of BBS, both English and Urdu, was then tested. Ten participants were requested to fill out the questionnaires. After the survey, the opinions of the participants were obtained regarding the survey questions, and they were asked what they understood from it. The ability of the participant to fill out the questionnaire on their own was observed, and all participants were encouraged to note any problem with the used wordings, layout, or other instructions that were present. These findings were then evaluated by the expert committee.

#### 2.1.6. Stage 6: Submission and Appraisal of All the Written Reports by Developers/Committee

The final report was the documentation of the cross-cultural adaptation process and its submission along with all reports to the committee. The final Urdu translated version of BBS was then used in the survey study.

### 2.2. Statistical Analysis

Data analysis was entered and performed using IBM SPSS 21 (IBM Corp., Armonk, NY, USA) statistical software, and the *p*-value was set to be statistically significant at 0.05. The characteristics of the subjects were studied through the use of descriptive statistics. For categorical and continuous variables, we calculated frequencies and proportions; we also considered calculating means and standard deviations. The Spearman correlation coefficient was also used to show that the BBS-U, UPDRS, and ABC scores were all correlated together.

#### 2.2.1. Reliability

For testing the reliability of BBS-U, the scale was tested among 80 patients with a diagnosis of PD disease. As per the recommendations for the reliability studies, the sample size for the current study was calculated using G*Power calculator [32]. Two physical therapists, assessors (A) and (B), examined each enrolled patient separately using the BBS-U. Assessor (A) conducted the examination first, and assessor (B) assessed the participants several hours later on the same day to measure the inter-rater reliability of BBS-U. The patients were assessed twice by one assessor (i.e., assessor A) on the first day to measure intra-rater reliability of BBS-U. Further, participants were re-examined 2 weeks later to assess the test-retest reliability of BBS-U. Intra-class correlation coefficient (ICC) using two way mixed analysis of variance was used to assess the test-retest, inter-rater, and intra-rater reliability of the BBS score [33]. The values of ICC lie between 0 and 1. Reliability of the study can be poor, moderate, good, or excellent with values < 0.5, between 0.5–0.75, between 0.75–0.90, and >0.90, respectively [34,35]. Cronbach’s α was used to measure internal consistency of BBS-U. Acceptable values for Cronbach’s α have to be more than 0.70 [36]. Moreover, item-total correlation for the internal consistency of the scale was determined through use of Spearman’s correlation coefficient (r_s_) and was interpreted as having little or no relationship, fair, moderate, and excellent relationship with values < 0.25, 0.25–0.50, 0.50–0.75, and ≥0.75, respectively [37]. 

#### 2.2.2. Bland and Altman Plot

The Bland and Altman plot was used to measure the degree of within-subject variation and limits of agreement with 95% confidence intervals [38]. The Bland–Altman plot (B&A) is a graphical method for describing and quantifying agreement between two quantitative measurements through the use of limits of agreement from the mean difference [39]. The plot only defines the agreement intervals, not whether they are acceptable or not. B&A established agreement limits using a simple formula based on the mean and standard deviation of two measurements. The mean and standard deviation of two measurements are used to calculate these statistical limits. The B&A plot used a graph to see if differences and other things were normal [40].

#### 2.2.3. Floor and Ceiling Effect

During the analysis, the floor and ceiling effect was also calculated. These effects are considered to exist if >15% of the patients have obtained maximum or minimum score out of the possible total score [41]. 

#### 2.2.4. Validity

Translation and cultural adaptation of the scale was used to assess the content validity of the scale [42,43]. The construct validity of the scale was assessed through convergent validity that was calculated using Pearson correlation (r). Pearson correlation coefficient was interpreted as very weak correlation, weak correlation, moderate correlation, strong correlation, and very strong correlation with values of 0.00 to 0.19, 0.20 to 0.39, 0.40 to 0.69, 0.70 to 0.89, and 0.90 to 1, respectively [44,45]. It was hypothesized that there will be moderate to strong negative correlation between BBS-U and the Urdu version of Unified Parkinson’s Disease Rating Scale parts II and III (UPDRS-II and UPDRS-III), whereas a moderate to strong positive correlation will be found with the Urdu version of Activities-specific Balance Confidence Scale (ABCS).

#### 2.2.5. Factor Analysis

Factor analysis is a method for condensing a huge number of variables into a smaller number of factors. This method sets the largest common variance from all variables and transforms it to a single score [46]. The correlation between two variables is measured by factor loading. The amount of variance a given variable contributes to a factor is measured by factor loading. In standard error of measurement (SEM,) a factor loading of 0.7 or greater indicates that the factor extracts sufficient variance from the variable. Eigenvalues are also known as characteristic roots. For each factor’s eigenvalues, we can see how much of the total variance can be explained by that factor. The commonality column tells us how much of the total variance is explained by the first factor [47]. Suppose our first factor accounts for 68% of the total variance, and the second factor accounts for 32%. In other words, the factor score can also be referred to as the component score. Using this score, you can index all variables and perform further analysis on the data. This score can be standardized by multiplying a common term. At all times, we will treat all variables as if they are factor scores and treat them accordingly [48].

## 3. Results

### 3.1. Psychometric Testing

In the study, 80 participants with PD were selected on the basis of inclusion and exclusion criteria. Out of these 80 participants, 50 (63%) were male and 30 (37%) were female. The average age of the participants were 62.35 ± 5.74 years, and the average age of PD onset was 56.48 ± 5.04 years. The average disease duration was 5.95 ± 1.67 (Table 1).

#### 3.1.1. Reliability

The ICC value for intra-rater, inter-rater, and test-retest reliability of BBS-U score was found to be 0.95 (95% CI: 0.93–0.97), 0.99 (95% CI: 0.99–1.00), and 0.99 (95% CI: 0.99–1.00), respectively (Table 2). Internal consistency measured by Cronbach’s α was 0.80. The range of Kappa correlation coefficient for intra-rater, inter-rater, and test-retest reliability of each BBS-U item was 0.84–0.96, 0.77–0.93, and 0.66–0.95, respectively (Table 2 and Table 3). Spearman coefficient of correlation between each item score and BBS-U score revealed the strongest statistically significant correlation between BBS item 5 (transfers) and BBS score (r = 0.79, *p* < 0.001) and the weakest non-significant relationship between BBS item 2 (standing unsupported) and BBS score (r = 0.16, *p* > 0.05). The range of correlation between each item score and BBS-U score was 0.16 to 0.79 (Table A2, Appendix B).

The Pearson correlation coefficient (r) for the clinical variables and BBS revealed that there was no correlation between age and BBS (r = 0.06, *p* = 0.61), age at onset and BBS (r = 0.06, *p* = 0.62), or disease duration and BBS (r = 0.18, *p* = 0.11) (Table 4).

#### 3.1.2. Factor Analysis

The dimensionality of any instrument is determined by using factor analysis [49,50]. Factor analysis of BBS revealed two factors when a criterion of parallel analysis was used. Total matrix variance for both factors was 47% (factor 1, 34.67%; factor 2, 12.20%). Factor 1 was related to dynamic activities, whereas factor 2 involved mostly static activities (Table 5).

#### 3.1.3. Floor and Ceiling Effect

No floor and ceiling effects were observed in the analysis of BBS-U. The percentage of respondents obtaining the highest score was 12.5% (Table A1, Appendix A). 

#### 3.1.4. Validity

The BBS-U scores showed a moderate negative correlation with UPDRS-II and III (r = −0.68, *p* < 0.001 and r = −0.78, *p* < 0.001, respectively). In contrast, there was a moderate positive correlation between BBS and ABCS (r = −0.53, *p* < 0.001) (Table 6).

**Figure 2:**
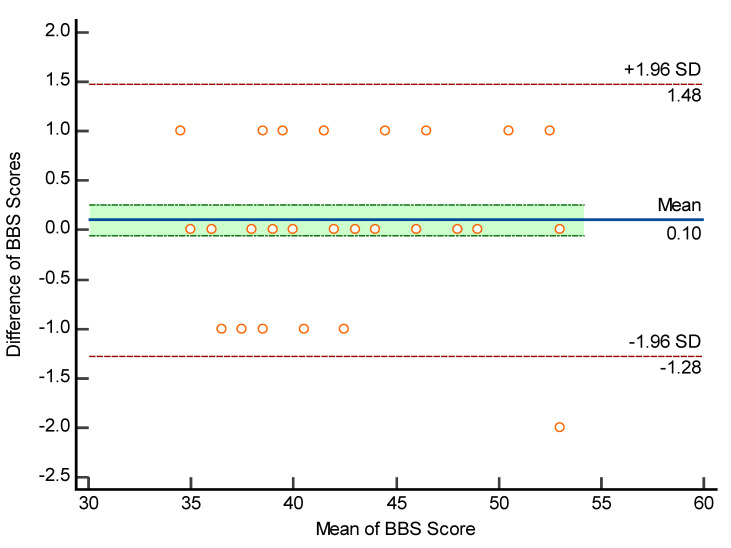
Bland–Altman plot for assessing the limits of agreement and within subject variation. ◦ indicates the differences in the each pair value of rater 1 and rater 2 on the vertical axis versus the averages of the each pair value (rater1 + rater2)/2) on the horizontal axis.

Figure 2 shows the Bland–Altman plot indicating within-subject variation and limits of agreement. An SD of ±1.96 was obtained, which indicates 95% limits of agreement, therefore strongly recommending the ICC values obtained.

## 4. Discussion

Over the last decade, many new tools have been developed for the assessment of balance function among the geriatric population as well as for patients with Parkinson’s disease [51,52,53]. BBS is a well-accepted assessment tool among clinical therapists, with outstanding reliability and validity [54]. The scale is relatively easy to administer as it requires less than fifteen minutes to administer; it also has been tested for its correlation with other disease-specific clinical tools, an important characteristic that merits it preference over other instruments [55,56]. 

In the study, 80 participants with PD were selected on the basis of inclusion and exclusion criteria. The current study had a higher proportion of male participants than females. It has also been reported that Pakistani men are disproportionately affected by PD, representing 63% of the affected population [57,58]. Furthermore, this is comparable to previous studies in which more males were recruited [23,24,25]. In the study conducted for the psychometric testing of the Japanese version, slightly more females than males were enrolled [18].

The current study found excellent inter-rater and test-retest reliability of BBS-U. The findings are comparable to those of other language versions of BBS, such as Japanese, Brazilian, and Persian [18,21,23]. Internal consistency measured by Cronbach’s α came out to be 0.80, whereas the α values were slightly higher in the Brazilian version (Cronbach’s α = 0.92), the Italian version (0.89) [25], and in the Iranian version (0.92) [24]. 

According to the findings of the present study, the Pearson correlation coefficient for clinical variables and BBS revealed that there was no correlation between BBS-U, age, and disease duration. The total BBS scores were not significantly correlated with the age of the patients in a validation study conducted in 2005 [21]. Nevertheless, these findings differ from those reported by other studies. A significant negative correlation was found between BBS and disease duration in the Brazilian version [23]. Similarly, in the Persian version, there was a significantly negative correlation between age and the total BBS score (r = −0.546, *p* < 0.001) reported [24]. 

The dimensionality of BBS-U was determined by using factor analysis as recommended [49,50]. It was carried out through varimax rotation. The factor structure of BBS-U was assessed through factor analysis. The value of the Kaiser–Meyer–Olkin measure (KMO) measure of sampling adequacy was 0.665 and Bartlett’s test of sphericity was significant (χ^2^ = 1059.62, *p* < 0.001). Factor analysis of BBS revealed two factors when a criterion of parallel analysis was used. Total matrix variance for both factors was 47% (factor 1, 34.67%; factor 2, 12.20%). Factor 1 was related to dynamic activities, whereas factor 2 involved mostly static activities. Similar factors were also reported in the Brazilian (factor 1 with 37.1% of total variation and factor 2 with 27.4% of total variation) [23] and the Persian versions (42.5 and 26.2% for factors 1 and 2, respectively) [24]. However, Taghizadeh and colleagues found that there was only one factor, with Kaiser–Meyer–Olkin = 0.92; Bartlett’s sphericity test, *p* < 0.001 [21].

This study found a moderate negative correlation between the UPDRS sections II and III and the Urdu version of BBS, with a *p* value < 0.001. Similar findings were reported in a study by Taghizadeh et al., in which the r_s_ value for UPDRS-II was −0.62 [21]. In another validation study by Qutubuddin et al., BBS scores were negatively associated (r_s_ = −0.58) with the most recent UPDRS motor examination [26]. A moderate positive correlation was found between BBS-U and ABCS (r_s_ = −0.55, *p* < 0.001). The correlation between BBS and ABCS has never been studied before. BBS-U analysis did not reveal a floor or ceiling effect. Studies have reported ceiling effects that may lead to an incorrect assessment of patients with mild deficits [26,59,60].

## 5. Conclusions

Our study revealed that the Urdu version of the BBS is a reliable and valid tool with excellent psychometric properties for the assessment of Urdu-speaking patients with PD. This tool may be used in future research projects throughout the world because it correlates well with other disease-specific scales in the Urdu-speaking population. Thus, obtaining accurate information about the balance status of people with PD, particularly during the drug off-phase, can assist in developing a more efficient treatment plan, and thus, BBS-U can be beneficial in this regard. 

## 6. Limitations

The main limitations of our study are related to the relatively low sample size, the heterogeneous sample, and the inclusion of only mild-to-moderate severity of PD (stage 1–3 of the modified Hoehn and Yahr scale). Stage IV and V patients were excluded from this study. As a result, our findings do not address the reliability of the BBS-U in patients with advanced PD. Another limitation of the current study is that the participants did not represent older people living in residential care facilities or those with orthopaedic or neurological disorders. These flaws should be considered for the generalizability of our findings and addressed in future studies. As noted, the reliability and validity of the BBS-U have only been assessed in the on-drug phase of PD patients, which is also a limitation of the current study. Furthermore, Rasch analysis was not performed due to the small sample size of the study. 

## Figures and Tables

**Table 1:** Demographics and clinical characteristics of the study subjects (*N* = 80).

Variables	Number (%)	Mean ± SD
Gender		
Male	50 (63)	
Female	30 (37)	
Educational level		
Cannot read and write	19 (23.75)	
Primary education (1–8)	21 (26.25)	
Secondary education (9–12)	25 (31.25)	
Above secondary education	15 (18.75)	
Current status employment		
Yes	07 (8.75)	
No	32 (40)	
Retired	41 (51.25)	
Age (years)		62.35 ± 5.74
Age of PD onset (years)		56.48 ± 5.04
Disease duration (years)		5.95 ± 1.67
ABCS		59.73 ± 3.84
BBS baseline		43.67 ± 5.60
UPDRS-II		25.38 ± 4.29
UPDRS-III		34.50 ±6.91

SD; standard deviation, PD; Parkinson’s disease, ABCS; Activities-specific Balance Confidence Scale, BBS; Berg Balance Scale, UPDRS; unified Parkinson’s disease rating scale.

**Table 2:** Inter-rater and intra-rater reliability (Kappa correlation coefficient) of each BBS-U item (*N* = 80).

	Inter-Rater Reliability				Intra-Rater Reliability			
Items	Rater-1	Rater-2	ICC(Lower–Upper)	Kappa	First Assessment	Second Assessment	ICC(Lower–Upper)	Kappa
1	3.50 ± 0.60	3.53 ± 0.55	0.89(0.83–0.93)	0.86(0.76–0.97)	3.50 ± 0.60	3.48 ± 0.59	0.89(0.84–0.93)	0.86(0.75–0.96)
2	3.50 ± 0.55	3.48 ± 0.55	0.96(0.94–0.97)	0.93(0.85–1.00)	3.50 ± 0.55	3.46 ± 0.55	0.94(0.91–0.96)	0.95(0.89–1.02)
3	3.53 ± 0.55	3.50 ± 0.55	0.96(0.94–0.97)	0.88(0.78–0.98)	3.53 ± 0.55	3.46 ± 0.55	0.90(0.85–0.94)	0.95(0.89–1.02)
4	3.58 ± 0.50	3.55 ± 0.50	0.95(0.92–0.97)	0.87(0.76–0.98)	3.58 ± 0.50	3.60 ± 0.52	0.81(0.71–0.87)	0.95(0.84–1.06)
5	3.25 ± 0.83	3.28 ± 0.84	0.98(0.97–0.99)	0.87(0.77–0.96)	3.25 ± 0.83	3.16 ± 0.80	0.94(0.91–0.96)	0.96(0.91–1.01)
6	3.33 ± 0.85	3.28 ± 0.87	0.97(0.95–0.98)	0.82(0.71–0.94)	3.33 ± 0.85	3.43 ± 0.76	0.79(0.70–0.86)	0.92(0.84–1.00)
7	3.18 ± 0.84	3.15 ± 0.83	0.98(0.97–0.99)	0.90(0.82–0.98)	3.18 ± 0.84	3.16 ± 0.80	0.95(0.93–0.97)	0.96(0.91–1.01)
8	2.85 ± 0.73	2.88 ± 0.72	0.98(0.96–0.98)	0.89(0.79–0.98)	2.85 ± 0.73	2.86 ± 0.67	0.94(0.90–0.96)	0.96(0.90–1.02)
9	2.83 ± 0.90	2.88 ± 0.82	0.97(0.95–0.98)	0.89(0.80–0.98)	2.83 ± 0.90	2.86 ± 0.91	0.93(0.89–0.96)	0.93(0.86–0.99)
10	3.13 ± 0.85	3.13 ± 0.85	1.00(1.00–1.00)	0.86(0.76–0.96)	3.13 ± 0.85	3.09 ± 0.80	0.94(0.90–0.96)	1.00(1.00–1.00)
11	2.78 ± 0.89	2.80 ± 0.91	0.99(0.98–0.99)	0.80(0.70–0.91)	2.78 ± 0.89	3.00 ± 0.89	0.76(0.65–0.84)	0.84(0.74–0.94)
12	2.73 ± 0.84	2.78 ± 0.83	0.97(0.95–0.98)	0.87(0.77–0.96)	2.73 ± 0.84	2.81 ± 0.83	0.94(0.91–0.96)	0.93(0.85–1.00)
13	3.08 ± 0.82	3.13 ± 0.82	0.96(0.95–0.98)	0.89(0.80–0.97)	3.08 ± 0.82	3.18 ± 0.76	0.89(0.83–0.93)	0.93(0.85–1.00)
14	2.30 ± 0.68	2.25 ± 0.74	0.95(0.93–0.97)	0.77(0.65–0.90)	2.30 ± 0.68	2.41 ± 0.69	0.87(0.80–0.91)	0.92(0.79–1.04)
Total	43.68 ± 5.60	43.58 ± 5.58	0.99(0.988–0.995)	0.95(0.90–1.00)	43.68 ± 5.60	43.96 ± 4.91	0.95(0.93–0.97)	0.90(0.79–1.01)

ICC: intra-class correlation coefficient.

**Table 3:** Test-retest reliability (Kappa correlation coefficient), internal consistency (Cronbach’s α), and item-total correlation (Spearman’s correlation coefficient (rs)) for BBS-U (*N* = 80).

Items BBS-U	Measure-ment-1	Measure-ment-2	SEM	SDC	ICC(Lower–Upper)	Kappa	α	Item to Total Correlation
1	3.50 ± 0.60	3.53 ± 0.55	0.20	0.56	0.77(0.67–0.85)	0.72(0.58–0.86)	NA	0.50
2	3.50 ± 0.55	3.48 ± 0.55	0.11	0.31	0.90(0.84–0.93)	0.88(0.78–0.98)	NA	0.06
3	3.53 ± 0.55	3.50 ± 0.55	0.11	0.31	0.86(0.78–0.91)	0.83(0.71–0.95)	NA	0.72
4	3.58 ± 0.50	3.55 ± 0.50	0.11	0.31	0.76(0.65–0.84)	0.95(0.88–1.02)	NA	0.69
5	3.25 ± 0.83	3.28 ± 0.84	0.11	0.31	0.93(0.89–0.95)	0.83(0.72–0.93)	NA	0.74
6	3.33 ± 0.85	3.28 ± 0.87	0.15	0.42	0.77(0.66–0.85)	0.74(0.61–0.87)	NA	0.60
7	3.18 ± 0.84	3.15 ± 0.83	0.11	0.31	0.93(0.89–0.96)	0.86(0.77–0.96)	NA	0.23
8	2.85 ± 0.73	2.88 ± 0.72	0.11	0.31	0.91(0.86–0.94)	0.84(0.72–0.95)	NA	0.30
9	2.83 ± 0.90	2.88 ± 0.82	0.16	0.45	0.89(0.83–0.93)	0.81(0.71–0.92)	NA	0.16
10	3.13 ± 0.85	3.13 ± 0.85	0.00	0.00	0.94(0.90–0.96)	0.86(0.76–0.96)	NA	0.23
11	2.78 ± 0.89	2.80 ± 0.91	0.11	0.30	0.63(0.47–0.74)	0.66(0.52–0.79)	NA	0.41
12	2.73 ± 0.84	2.78 ± 0.83	0.16	0.44	0.96(0.93–0.97)	0.90(0.82–0.99)	NA	0.59
13	3.08 ± 0.82	3.13 ± 0.82	0.16	0.43	0.84(0.76–0.89)	0.81(0.70–0.92)	NA	0.42
14	2.30 ± 0.68	2.25 ± 0.74	0.15	0.41	0.82(0.73–0.88)	0.72(0.59–0.85)	NA	0.70
Total	43.68 ± 5.60	43.58 ± 5.58	0.50	1.39	0.99(0.99–1.00)	0.97(0.91–1.03)	0.81	NA

ICC: intra-class correlation coefficient, CI: confidence interval, SEM: standard error of measurement, SDC: smallest detectable change, NA: not applicable.

**Table 4:** Pearson correlation coefficient (r) and *p*-value between demographic, clinical variables, and BBS-U.

Variables	r	*p*	Classification
Age (years)	0.06	0.61	No correlation
Age at onset (years)	0.06	0.62	No correlation
Duration (years)	0.18	0.11	No correlation

**Table 5:** Factor analysis of BBS-U.

BBS-Item No.	Statement	Components	
		1 (Dynamic)	2 (Static)
BBS1	Sitting to standing	0.65	
BBS2	Standing unsupported		0.64
BBS3	Sitting unsupported	0.79	
BBS4	Standing to sitting	0.78	
BBS5	Transfers	0.83	
BBS6	Standing with eyes closed	0.73	
BBS7	Standing with feet together		0.38
BBS8	Reaching forward with outstretched arm		−0.41
BBS9	Retrieving object from floor		−0.38
BBS10	Turning to look behind		0.70
BBS11	Turning 360 degrees	0.53	−0.46
BBS12	Placing alternate foot on stool	0.65	
BBS13	Standing with one foot in front	0.58	
BBS14	Standing on one foot	0.82	
Total		4.85	1.72
Initial eigenvalues (% of variance)		34.67	34.67

**Table 6:** Pearson’s correlation coefficient (r) and *p*-value for investigating construct validity of BBS-U using section II and III of Unified Parkinson’s Disease Rating Scale (UPDRS-II, and UPDRS–III) and Activities-specific Balance Scale (ABCS).

Variables	r	*p*	Classification
UPDRS-II	−0.68 **	<0.001	Moderate correlation
UPDRS-III	−0.78 **	<0.001	Moderate correlation
ABCS	0.53 **	<0.001	Moderate correlation

** *p* < 0.01.

## Data Availability

The data generated or analyzed during this study are presented in this article, and further enquiries can be directed to the corresponding author.

## Appendix A

**Table A1:** Floor and ceiling effects of BBS-U.

BBS-U	Measurement-1	Lowest Score	Highest Score	Missing Scores	Floor Effect (%)	Ceiling Effect (%)
Total score	43.68 ± 5.60	35	53	0	0	12.5%

## Appendix B

**Table A2:** Correlation coefficient of each item of BBS-U with total points.

Item No	Statement	p	r_s_
BBS1	Sitting to standing	<0.001	0.58 **
BBS2	Standing unsupported	0.16	0.16
BBS3	Sitting unsupported	<0.001	0.76 **
BBS4	Standing to sitting	<0.001	0.75 **
BBS5	Transfers	<0.001	0.79 **
BBS6	Standing with eyes closed	<0.001	0.68 **
BBS7	Standing with feet together	<0.001	0.38 **
BBS8	Reaching forward with outstretched arm	<0.001	0.40 **
BBS9	Retrieving object from floor	<0.001	0.31 **
BBS10	Turning to look behind	<0.001	0.28 **
BBS11	Turning 360 degrees	<0.001	0.55 **
BBS12	Placing alternate foot on stool	<0.001	0.70 **
BBS13	Standing with one foot in front	<0.001	0.53 **
BBS14	Standing on one foot	<0.001	0.73 **

** *p* < 0.01.
